# Activation of central Angiotensin-(1-7)/Mas receptor alleviates synaptic damage in diabetes-associated cognitive impairment via modulating AKT/FOXO1/PACAP axis

**DOI:** 10.7150/ijbs.99617

**Published:** 2025-04-09

**Authors:** Sai Tian, Tianyu Wu, Zhou Zhang, Shanshan Lv, Xinlu Ji, Zhicong Zhao, Xuelin Ma, Jin Wang, Yan Bi

**Affiliations:** 1 Department of Endocrinology, Endocrine and Metabolic Disease Medical Center, Nanjing Drum Tower Hospital, Affiliated Hospital of Medical School, Nanjing University, Nanjing China.; 2 Branch of National Clinical Research Centre for Metabolic Diseases, Nanjing, China.

**Keywords:** Angiotensin-(1-7)/Mas receptor, Synaptic damage, Pituitary adenylate cyclase-activating polypeptide, Diabetes-associated cognitive impairment

## Abstract

Synaptic damage is a pathological hallmark of diabetes-associated cognitive impairment (DACI). Angiotensin-(1-7)/Mas receptor has been implicated in regulating peripheral glucose homeostasis and exerting neuroprotective effects on central nervous system. This study investigated the possible roles of Angiotensin-(1-7)/Mas receptor in DACI, aiming to elucidate the molecular mechanisms underlying synaptic damage. We observed a significant reduction of Angiotensin-(1-7) levels in type 2 diabetic patients with mild cognitive impairment and diabetic cognitive impairment mice. Downregulation of Angiotensin-(1-7)/Mas receptor was associated with the decreased synaptic protein expressions in diabetic cognitive impairment mice, and high glucose-stimulated primary hippocampal neurons. Administration of the Mas agonist AVE 0991 into the hippocampus effectively ameliorated synaptic and memory dysfunctions in diabetic cognitive impairment mice. Inhibition of hippocampal neuronal Mas receptor aggravated synaptic damage. Mechanistically, we first elucidated that pituitary adenylate cyclase-activating polypeptide (PACAP) serves as a downstream synaptic function-related target gene of Mas receptor. Furthermore, we identified AKT/FOXO1 pathway as a critical downstream mediator of Mas receptor in modulating PACAP expression, with FOXO1 binding directly to the PACAP promoter region. In conclusion, Angiotensin-(1-7)/Mas receptor may modulate synaptic function-related target gene PACAP expression through AKT/FOXO1 pathway, thereby providing a deeper theoretical basis and molecular target for future DACI treatment.

## Introduction

Diabetes mellitus has become a global public health concern due to its increasing prevalence. Cognitive impairment is a common complication of diabetes, affecting patients' daily life, learning abilities, and social abilities 1. Clinical trials indicate that glycemic control has limited efficacy in improving cognitive function 2, underscoring the need for innovative preventive and therapeutic strategies against diabetes-associated cognitive impairment (DACI).

Hippocampal synaptic plasticity is integral to the formation of learning and memory 3-5, and synaptic damage is an early pathological hallmark of cognitive impairment 6-8. Our previous research revealed that a mice model exhibiting obesity-related insulin resistance, alongside mild hyperglycemia, can induce synaptic damage and consequent cognitive impairment 9. Further exploration of the mechanisms underlying DACI may unveil novel intervention targets to alleviate synaptic damage and ameliorate disease progression.

Angiotensin-(1-7)/Mas receptor [ANG-(1-7)/MasR] axis has been shown to exert neuroprotective effects on the central nervous system, by modulating inflammation, oxidative stress, autophagy, and apoptosis 10-13. The receptor Mas, a G protein-coupled receptor encoded by the oncogene Mas1, is highly expressed in the brain and selectively binds to ANG-(1-7), a key effector peptide of the non-classical RAS 14, 15. ANG-(1-7)/MasR has also been reported to increase glucose utilization and reduce insulin resistance in the periphery 16-18. However, the role of ANG-(1-7)/MasR in DACI, particularly the precise mechanisms involved in synaptic damage, remains unclear.

In this study, we identified a significant reduction of ANG-(1-7)/MasR in DACI. In addition, we found that MasR may modulate the expression of synaptic function-related target genes through AKT/FOXO1 pathway, thereby regulating synaptic function. This investigation may provide a deeper theoretical foundation and molecular targets for future DACI treatment.

## Materials and methods

### The clinical cross-sectional study

#### Study population

This cross-sectional study was conducted from October 2022 to December 2023 at Drum Tower Hospital affiliated to Nanjing University Medical School. We recruited hospitalized patients with type 2 diabetes mellitus (T2DM) aged 50 to 75 years with diabetes history > 3 years. Nanjing Drum Tower Hospital Ethics Committee granted ethical approval for this study (Approval Numbers: 2022-332-02, Clinical Trials: NCT 05590442). The work was performed in accordance with the Code of Ethics of the World Medical Association (Declaration of Helsinki) for experiments involving humans.

#### Clinical data collection

The following demographic and physical characteristics were collected: age, sex, education years, diabetes duration, height, weight, waist circumference, and resting blood pressure. After at least 8 h of overnight fasting, blood samples were obtained to determine fasting blood-glucose (FBG), fasting insulin (FINS), fasting C peptide (FCP), glycosylated hemoglobin (HbA1c), and lipid profiles (triglycerides [TG], total cholesterol [TC], low-density lipoprotein cholesterol [LDL-c], and high-density lipoprotein cholesterol [HDL-c]).

#### Measurement of serum angiotensin-(1-7) levels

Serum samples from hospitalized patients were collected at 7 AM following an overnight fast. Serum Angiotensin-(1-7) were determined by Human enzyme-linked immunosorbent assay (ELISA) kits (Cusabio Biotech, Wuhan, China).

#### Cognitive assessments

All participants underwent Montreal Cognitive Assessment (MoCA) (Beijing version) 19 by a well-trained examiner. MoCA, a highly sensitive screening tool of MCI, used to divide our T2DM patients into mild cognitive impairment (MCI) (MoCA < 26) and non-MCI (MoCA ≥ 26).

#### Magnetic resonance imaging (MRI) imaging data acquisition and preprocessing

All participants underwent high-resolution three-dimensional T1 weighted imaging (3D-T1WI) using Philips 3 T MRI scanners (TR=8.10 ms, TE=3.70 ms, feld of view = 256 × 256 × 192 mm^3^, voxel size=1×1×1 mm^3^). The subcortical nuclei, including the thalamus, caudate, putamen, pallidum, hippocampus, amygdala, and accumbens, were automatically delineated using FreeSurfer version 6.0.0 image analysis software (http://freesurfer.net/). Subsequently, the hippocampus was further subdivided into 12 distinct regions: the hippocampal tail, subiculum, CA1, fissure, presubiculum, parasubiculum, molecular layer, dentate gyrus, CA2/3, CA4, fimbria, and HATA. Additionally, the estimated total intracranial volume (TIV) was extracted to adjust for variations in head size.

### In vitro and in vivo experiments

#### Mouse model

The animal experiment protocols were approved by the Laboratory Animal Care Guidelines of Nanjing University. Eight-week-old male mice were fed a high-fat diet (HFD, 60% fat, Research Diets, USA) for 24 weeks. As control, age-matched male mice were fed a standard normal diet (Chow) for the same duration. Fasting blood glucose levels were measured from tail-vein blood after an overnight fast using the glucometer (Contour TS, Bayer).

##### Glucose tolerance test (GTT) and insulin tolerance test (ITT)

For GTT, basal blood glucose levels of mice were measured, followed by intraperitoneal injection with 2g/kg weight glucose after a 12-h fast. For ITT, basal blood glucose levels of mice were measured, followed by intraperitoneal injection with insulin (0.5 unit/kg body weight) after a 4-h fast. Blood glucose levels were recorded after 15, 30, 60 and 120 min using blood obtained via the tail vein.

#### Cannulas implantation and stereotaxic injections

The animals were immobilized in a stereotactic apparatus with their heads positioned flat, and holes were drilled in the skull for the insertion of cannula (RWD Life Science, Shenzhen, China). The cannula was carefully placed within 1 mm above the target hippocampal region (location: anteroposterior, 2.4 mm; mediolateral, ± 2.2 mm; dorsoventral, - 2.5 mm), and was secured with three skull screws before being further stabilized with acrylic dental cement. The Mas agonist AVE 0991 was dissolved in DMSO. A total of 0.5 μL AVE 0991 (0.3mg/kg/day, MedChemExpress) or Vehicle (0.1% DMSO) was microinjected into the hippocampal region over a period of 5 minutes using the Hamilton syringe driven by a microinfusion pump, continuously for 2 weeks. Following each injection, the injection cannula was kept in place within the guide cannula for an additional 1 minute to ensure no leakage of the injected liquid.

#### Viral injection

Adeno-associated virus (AAV) adenoviruses expressing short-hairpin RNA (shRNA) specific for neuronal MasR or scrambled shRNA were infused into the bilateral hippocampal region. The virus (titer: 5e+12 viral genomes/mL) was infused at a rate of 200 nL/ min (1.0 µl/side) followed by a 10-min of rest.

#### Morris water maze (MWM)

The MWM tests were used to evaluate memory functions. Briefly, the MWM test comprised three stages, a visible platform test (day 1), followed by a hidden platform test (day 1- day 5), and a probe trial (day 6). During the hidden platform training, mice were trained pseudorandomly and equally to find the hidden platform within 60s in each of the 4 quadrants once a day. The latency, reflecting the time each mouse required to locate the platform, was recorded (MobileDatum Co. Ltd, Shanghai, China). During the probe trial (without a platform), mice were placed in the quadrant opposite to the platform to swim for 60s. The number of platform crossings, as well as the percentage of total time spent in the target quadrant were recorded (MobileDatum Co. Ltd, Shanghai, China).

#### Mouse MRI data acquisition and analysis

We used a 9.4 T Bruker MR system to obtain MRI data at the Department of Radiology, the Affiliated Drum Tower Hospital of Nanjing University Medical School. Mice were anesthetized with isoflurane and positioned within the fixed head setup using the mouse coil. T2-weighted anatomical scans and diffusion tensor imaging (DTI) data were collected, and fractional anisotropy (FA) were generated. The normalization of the mean value of the control group was defined through data from chow diet-fed mice. Data analysis was conducted by the Institute of High Energy Physics, Chinese Academy of Sciences.

#### RNA sequencing (RNA-Seq) and analysis

Total RNA from mouse hippocampal tissue or primary hippocampal neuron cells was extracted. Oebiotech (Shanghai, China) and Novogene (Tianjin, China) performed the library preparation, clustering/sequencing and data analysis.

#### Mice immunofluorescence staining

Tyramide Signal Amplification (TSA) technology was used for immunofluorescence staining of mice brain tissue sections. We used a Pannoramic Digital Slide Scanner (MIDI II, 3D HISTECH) to capture immunofluorescence images, and a Tissue-FAXS Viewer software (Tissue Gnostics) to view the images.

#### Transmission electron microscope (TEM)

We isolated and sectioned the mice hippocampus into 1 mm^3^ pieces. These tissue samples were then fixed overnight at room temperature in a solution containing 0.1 M sodium cacodylate buffer and 2.5% glutaraldehyde. After fixation, the specimens were rinsed with 0.1 M PBS and subsequently postfixed in 1% osmium tetroxide for 1 hour in darkness. Following postfixation, the samples underwent three washes with 0.1 M phosphate buffer, each for 15 minutes. Subsequently, the specimens were dehydrated through a series of ethanol concentrations (50%, 70%, 80%, 90%, 95%, and 100%) for 15 minutes at each step. Ultrathin sections of 80 nm were stained with uranyl acetate and lead acetate, and captured in a Hitachi H7650 electron microscope at 80 kV. Synapses were identified and quantified by the presence of synaptic vesicles and postsynaptic densities.

#### Enzyme-linked immunosorbent assay (ELISA)

ELISA assay was conducted to assess the concentrations of Angiotensin-(1-7) following the manufacturer's instructions provided with the Mouse Angiotensin-(1-7) ELISA Kit (Cusabio Biotech, Wuhan, China).

#### Western blot

We used RIPA lysis buffer (ThermoFisher Scientific, USA) to extract the proteins from tissues or cells, and a BCA protein assay kit (ThermoFisher Scientific, USA) to quantify their concentrations. We loaded 40 micrograms of proteins lysates onto 8% or 10% SDS-PAGE gels, and subsequently transferred these proteins from gels to polyvinylidene difluoride (PVDF) membranes (Millipore, USA). After blocking with either 5% bovine serum albumin (BSA) or 5% nonfat milk for 2 hours at room temperature, the membranes were incubated with the primary antibodies overnight at 4°C, and incubated with horseradish peroxidase (HRP)-conjugated secondary antibodies for 1 hour at room temperature. The protein bands were visualized via an ECL Detection Kit (Tanon Technologies, Shanghai, China), and the protein levels were quantified and analyzed by Image J software (NIH, USA).

#### Cell immunofluorescence staining

The cells cultured on the coverslips were washed with phosphate-buffered saline (PBS) 3 times, and then fixed with 4% paraformaldehyde (PFA) for 15 minutes. After fixation, they were incubated in PBS containing 5% BSA and 0.3% Triton-X 100 at room temperature for 1 hour. Subsequently, primary antibodies were applied and left to incubate overnight at 4°C. On the following day, appropriate fluorescent secondary antibodies were added and incubated at room temperature for 1 hour. The cells were then stained with DAPI for 10 minutes at room temperature. Finally, the Leica TCS SP8 confocal microscope was used to capture images.

#### Fluorescent image quantification

The images were converted to 8-bit format using Image J software to quantify the total gray value within the defined area. The mice brain sections were stained with synapsin I and postsynaptic density protein 95 (PSD 95) antibodies, and the gray values of them were calculated for each selected area (averaged over 5 areas per mouse). The primary neurons were stained with synapsin I and microtubule-associated protein 2 (MAP2) antibodies, and the puncta number-to-neurite length ratio in the designated area was computed.

#### RNA isolation and quantitative RT-PCR (qRT-PCR)

Trizol reagent (ThermoFisher Scientific, USA) was used to extract total RNA from tissue or primary hippocampal neuron cells, and Reverse Transcription Kit (Takara, Japan) was used to reverse-transcribed RNA into cDNA. Then, qRT-PCR was conducted using SYBR Green on Light Cycler 480 System (Roche, Switzerland). The amount of mRNA was normalized by the expression of β-actin. All primer sequences were provided in Supplementary Table.

#### RNA interference

GenePharma (GenePharma, Shanghai, China) synthesized the siRNA oligos. The designated siRNA was transfected into primary hippocampal neuron cells using Lipofectamine 3000 (Invitrogen). Cell samples were collected at 48 or 72 hours post-transfection, followed by RNA and protein extraction. The effectiveness of gene silencing was then assessed using qRT-PCR assays or Western blot analysis. The sequences of designated siRNA were listed in Supplementary Table.

#### Reagents and antibodies

AVE 0991 (cat no: HY-15778) was purchased from MedChemExpress (New Jersey, USA). PACAP (PACAP 1-38, cat no: S8415) was purchased from Selleck Chemicals (USA). The following antibodies were used: Neun antibody (cat no: ab177487) from Abcam; GFAP antibody (cat no: ab68428) from Abcam; Iba-1 antibody (cat no: 019-19741) from Wako; MAP2 antibody (cat no: ab5392) from Abcam; PSD 95 antibody (cat no: ab18258) from Abcam and (cat no: 3450) from Cell Signaling Technology; Synapsin I antibody (cat no: AB1543) from Millipore; Mas antibody (cat no: sc-390453) from Santa Cruz and (cat no: MB11189) from Bioworld; FOXO1 antibody (cat no: 2880) from Cell Signaling Technology and (cat no: 18592-1-AP) from Proteintech; AKT antibody (cat no: 4691) from Cell Signaling Technology; phospho-AKT antibody (cat no: 4060) from Cell Signaling Technology; β-Actin (cat no: AP0060) from Bioworld.

#### Primary neuron culture and treatments

The hippocampus was extracted from E14-16 mouse embryos, and after removal of the meninges, it was finely dissected into small fragments and subjected to a 30-minute incubation at 37°C in 0.025% trypsin. Afterward, the cell suspensions underwent filtration through a cell strainer, and were transferred to neurobasal medium supplemented with L-glutamine and B-27. Pre-coated poly-D-lysine (PDL) 6-well, 12-well, and 24-well plates, with plastic coverslips, were prepared. Cells were seeded accordingly: 24-well plates for synaptic staining and density quantification, 12-well plates for RNA extraction, and 6-well plates for protein extraction. Primary neurons were cultured for 7-10 days prior to RNA interference or AVE 0991 treatment (at concentrations of 10^-6^, 10^-7^, 10^-8^ M).

### Statistical analysis

SPSS 26.0 (SPSS Inc.,Chicago, IL) and GraphPad Prism 9.0 were used for statistical analyses. Continuous variables were presented as mean ± standard deviation (SD), mean ± standard error of mean (SEM), or median with 25th and 75th percentiles. Chi-squared test (χ^2^) was employed to compare the qualitative variables. The odds ratios (OR) and 95% confidence intervals (95% CI) for the risk factors of MCI in T2DM patients were estimated by using logistic regression models. With regard to MRI data, analyses of covariance (ANCOVA) were used to compare differences in brain structure, controlling for age, sex, education and estimated total intracranial volume. Partial correlation analysis was performed to evaluate the relationship between serum Angiotensin-(1-7) level and brain hippocampus subfield structure. Data were analyzed using one-way ANOVA, two-way ANOVA, or unpaired two-tailed t test. A p-value less than 0.05 was considered statistically significant.

## Results

### Reduced serum ANG-(1-7) levels are associated with cognitive decline in type 2 diabetic patients with mild cognitive impairment

**Figure 1:**
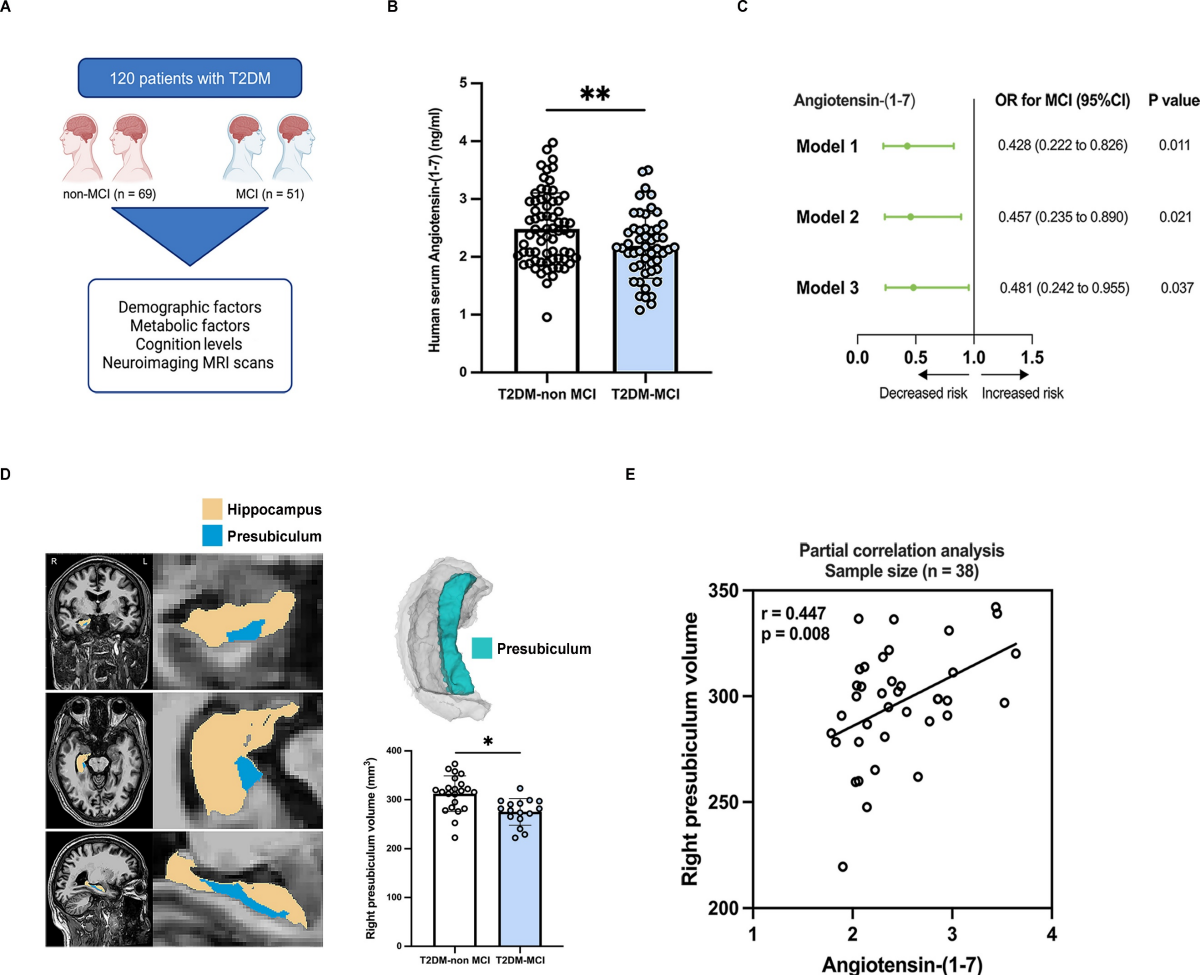
** Reduced serum ANG-(1-7) levels are associated with cognitive decline in type 2 diabetic patients with mild cognitive impairment.** (A) Clinical scheme of 120 subjects with T2DM. (B) Serum levels of Angiotensin-(1-7) in T2DM-non MCI and T2DM-MCI patients. (C) Logistic regression analyses producing odds ratios with 95% confidence intervals (OR, 95% CI) for MCI. Model 1: unadjusted; Model 2: adjusted for age, sex, and education; Model 3: adjusted for model 2 and FBG. (D) Left: An example of MRI hippocampal segmentation in coronal, axial, and sagittal views. Right: Volumes of right-presubiculum in T2DM-non MCI group and T2DM-MCI group, using analyses of covariance controlling for sex, age, years of education, and total intracranial volume. (E) Association between right-presubiculum volume and Angiotensin-(1-7) level, adjusted for age, sex, education, FBG, and estimated total intracranial volume. *p < 0.05, and **p < 0.01. Student's t-test (B), Logistic regression analysis (C), Covariance analysis (D) or Partial correlation analysis (E) were used for statistical analysis.

A total of 120 T2DM patients were recruited into this study, including 69 non-MCI controls and 51 MCI patients matched for age, sex and education level (**Figure 1A**). Compared with T2DM-non MCI, T2DM-MCI patients presented with elevated FBG and reduced serum Angiotensin-(1-7) levels (all p < 0.05; **Table 1 and Figure 1B**). No significant differences were found between the two groups in the levels of body mass index (BMI), waist-to-hip ratio (WHR), systolic blood pressure (SBP), diastolic blood pressure (DBP), FINS, FCP, HbAlc, the homeostasis model assessment of insulin resistance (HOMA-IR), TG, TC, HDL-c, LDL-c, serum creatinine and C-reactive protein (p > 0.05; **Table 1**). Moreover, T2DM-MCI patients scored lower on the MoCA scores than T2DM-non MCI controls (p < 0.05; **Table 1**). After adjustment for age, sex, education level and FBG, logistic regression analysis revealed that higher serum Angiotensin-(1-7) levels were associated with a lower risk of MCI in T2DM patients (OR = 0.481, p = 0.037; **Figure 1C**). Additionally, neuroimaging MRI scans were performed on 38 T2DM patients (22 non-MCI and 16 MCI patients). We focused on the hippocampus structure, a crucial role in cognitive functions. In this study, the analysis of hippocampal subregions revealed that the right-presubiculum volumes were smaller in MCI group than in T2DM-non MCI group after controlling for age, sex, education and estimated total intracranial volume (275.27 ± 6.73 mm^3^ vs 312.45 ± 7.78 mm^3^, p = 0.036; **Table 2, Figure 1D**). A previous large population-based cohort study reported that right-presubiculum volume was associated with the risk of dementia^20^. No significant differences were observed in the hippocampal tail, subiculum, CA1, fissure, parasubiculum, molecular layer, CA3, CA4, fimbria, HATA, and whole hippocampal volume between the two groups (all p > 0.05; **Table 2**). The partial correlation analysis revealed a positive correlation between the right-presubiculum volumes and serum Angiotensin-(1-7) levels in T2DM patients after adjusting for age, sex, education, FBG, and estimated total intracranial volume (r = 0.447, p = 0.008; **Figure 1E**).

### Diabetic mice exhibit cognitive impairment and synaptic damage in the hippocampal brain region

**Figure 2:**
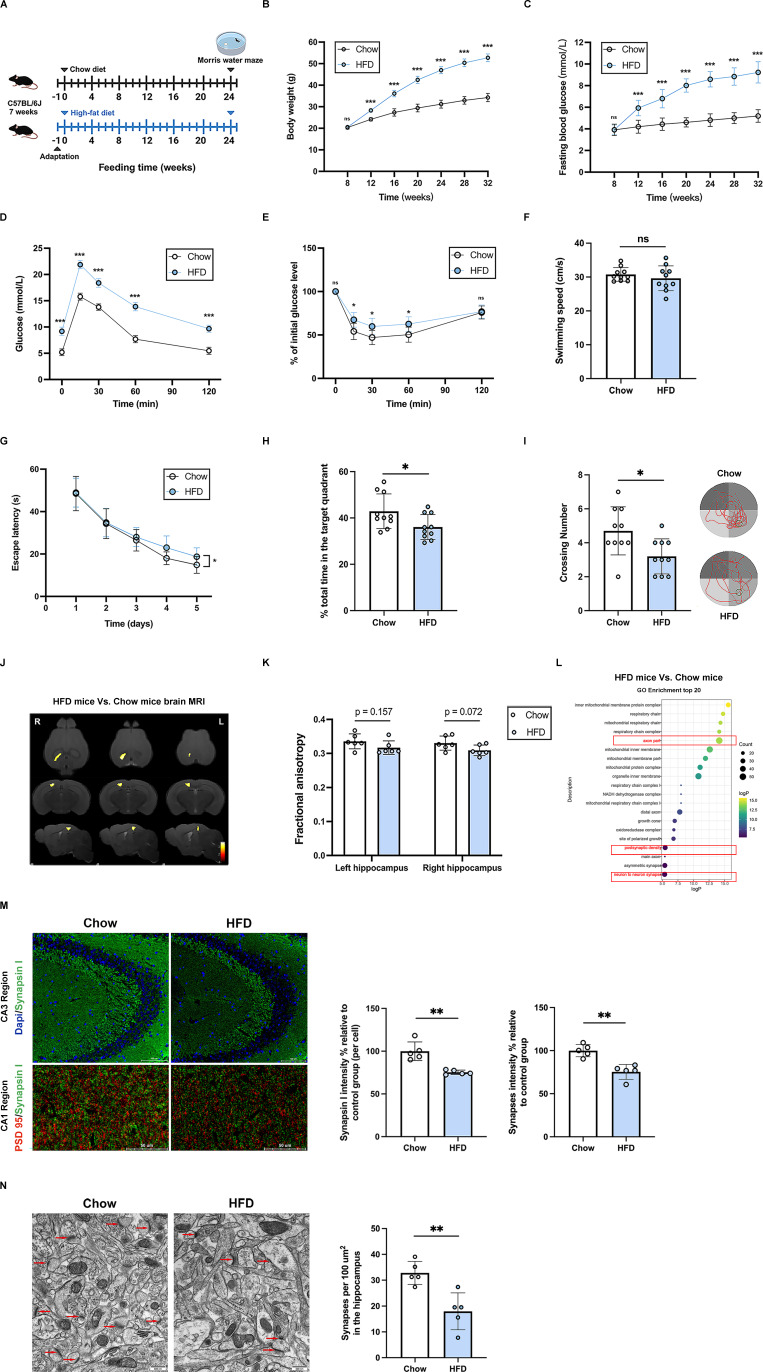
** Diabetic mice exhibit cognitive impairment and synaptic damage in the hippocampal brain region.** (A) Experimental scheme. (B-C) Changes in body weight and fasting blood glucose with feeding time in mice. (D-E) Glucose tolerance tests and insulin tolerance tests of mice for Chow-control group and HFD-induced diabetes group. (F) Average swimming speed of mice in the visible-platform test. (G) Time to find the hidden platform in the Morris water maze (escape latency) was analyzed at 32 weeks of age in mice for Chow-control group and HFD-induced diabetes group. (H) The percentage of time spent in the target quadrant out of the total test time during the probe trial in the Morris water maze for two groups of mice. (I) The numbers of platform crossings and route maps on the last day in the Morris water maze probe trial. (J) Heat maps generated from FA of DTI sequence acquired from HFD mice and Chow mice comparison in axial, coronal and sagittal views. (K) Differences of FA of DTI sequence in hippocampal region by magnetic resonance imaging (MRI) between HFD mice and Chow mice. (L) The top 20 terms of GO Enrichment analysis of RNA sequencing data from mouse hippocampal tissue comparing HFD mice and Chow mice. (M) Up: representative confocal images of hippocampal immunostaining for pre-synaptic marker Synapsin I (green) in hippocampus CA3 regions. Scale bars, 100 um. Down: representative confocal images depict synaptic staining for pre-synaptic marker Synapsin I (green) and post-synaptic marker PSD95 (red) in hippocampus CA1 regions. Scale bars, 50 um. Right: relative level of synaptic density. (N) Left: Representative electron microscopy of the synaptic structures in mouse hippocampus. Arrows indicate the synapses. Scale bar, 0.5 um. Right: Quantification of synaptic density in mouse hippocampus. Data are presented as the mean ± SD. n = 12 mice/group (B-C), n = 10 mice/group (F-I), n = 6 per group (D-E, J-K), n = 5 per group (M-N), n = 3 per group (L). *p < 0.05, **p < 0.01, and ***p < 0.001; ns, not significant. Unpaired t test (F, H-I, K, M-N) or two-way ANOVA (B-E, G) were used for statistical analysis.

A high-fat diet (HFD)-induced diabetic mice model was constructed by feeding C57BL/6 mice a HFD for 24 weeks (**Figure 2A**). By 32 weeks, mice in the HFD group exhibited significantly higher body weight and FBG levels than the Chow-control group (**Figure 2B-E**). MWM behavioral test was conducted to assess the spatial learning and memory function of the mice. Compared with the Chow-control mice, HFD-induced diabetic mice exhibited longer escape latencies in the hidden platform test, fewer number of platform crossings and less time in the target quadrant in the probe trial (**Figures 2F-I**). MRI/DTI can be used to evaluate white matter integrity, and FA is used to assess the microstructure of white matter. The FA values in the hippocampal brain region were reduced in HFD-induced diabetic mice compared to the Chow-control group, indicating microstructural damage in the hippocampal brain region of diabetic mice, but this difference did not reach statistical significance (**Figure 2J-K**). Additionally, the mice hippocampal tissues from two groups were obtained for RNA sequencing. GO enrichment analysis showed that the differentially expressed genes (DEGs) were primarily enriched in axon part, postsynaptic density, neuron to neuron synapse, and mitochondria-related proteins part (**Figure 2L**). Given the pivotal role of synaptic plasticity in memory function, immunofluorescence staining was used to observe synapses in hippocampal sections. The analysis of colocalized puncta of synapsin I and PSD 95 revealed fewer synapses in diabetic mice compared with Chow-control mice (**Figure 2M**). Moreover, TEM results exhibited a decrease in the number of synapses in the hippocampal region of diabetic mice (**Figure 2N**). Collectively, these results demonstrate that hippocampal synaptic damage is a fundamental pathological feature of cognitive impairment in diabetes.

### Downregulation of central ANG-(1-7)/MasR is implicated in synaptic damage in diabetic cognitive impairment

**Figure 3:**
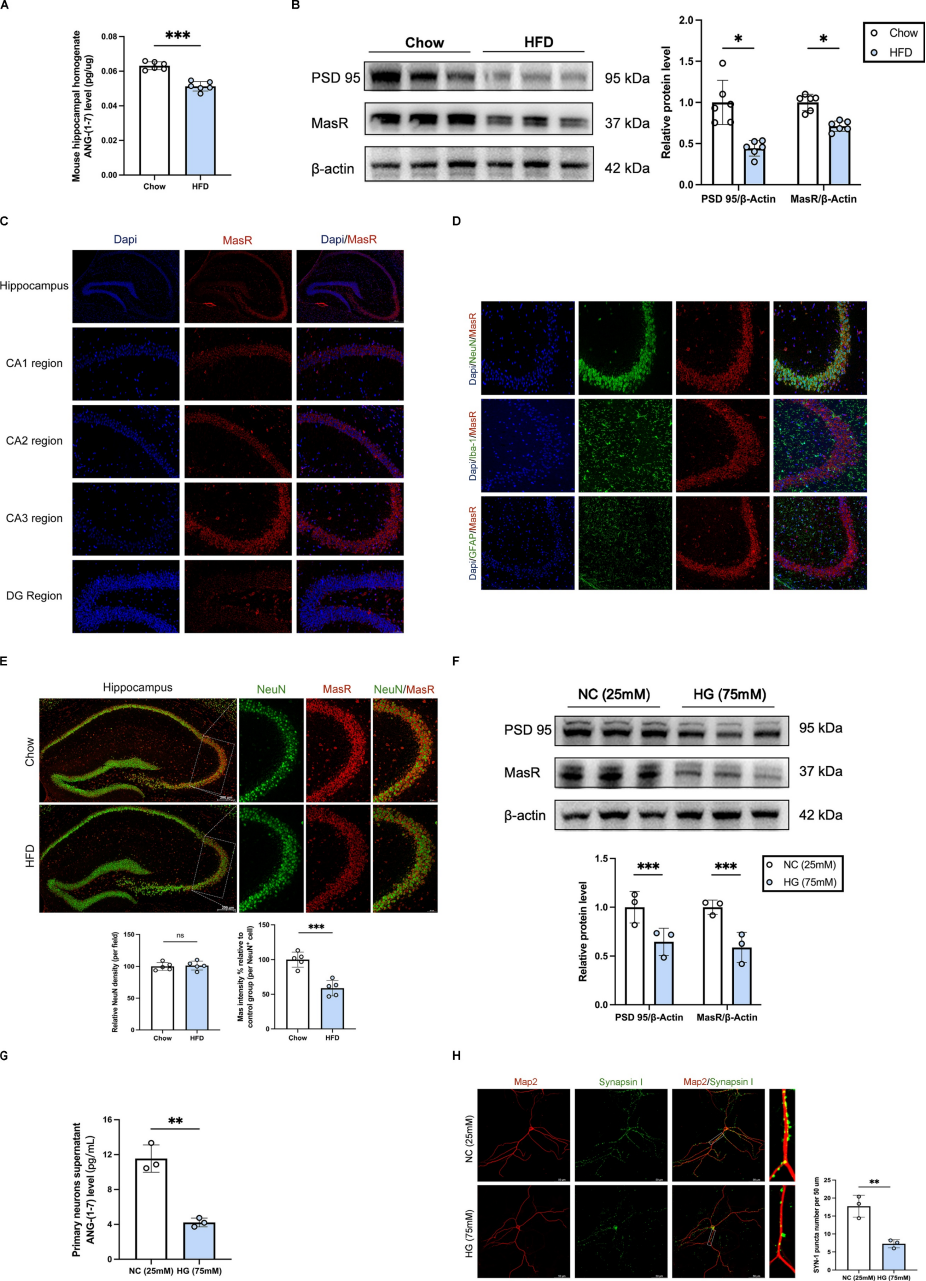
** Downregulation of central ANG-(1-7)/MasR is implicated in synaptic damage in diabetic cognitive impairment.** (A) Angiotensin-(1-7) levels in mouse hippocampal tissue homogenate between HFD mice and Chow mice. (B) MasR and PSD 95 relative protein expression levels in mouse hippocampal brain tissue between HFD mice and Chow mice. (C) MasR immunofluorescence staining in different subregions of the mouse hippocampus, including CA1, CA2, CA3 and DG subregion. Scale bars, 50 um. (D) MasR immunofluorescence staining on different nerve cells in the CA3 subregion of the mouse hippocampus, including neurons, microglia, and astrocytes. Scale bars, 50 um. (E) MasR immunofluorescence staining on neurons in the CA3 subregion of the mouse hippocampus between HFD mice and Chow mice. Scale bars, 50 um. (F) MasR and PSD 95 relative protein expression levels in primary hippocampal neuron cells from C57BL/6 mice hippocampus cultured with the negative control (NC, 25mM) medium or high glucose (HG, 75mM) medium. (G) Angiotensin-(1-7) levels in the supernatant of the primary hippocampal neuron cells between the NC (25mM) group and HG (75mM) group. (H) Left: representative confocal images of MAP2 (red) and Synapsin I (green) in primary hippocampal neuron cells. Right: quantification of Synapsin I-positive puncta per 50 um of MAP2-positive neurites between NC (25mM) group and HG (75mM) group. Scale bars, 50 um. Data are presented as the mean ± SD. n = 6 mice/group (A-B), n = 5 per group (C-E), n = 3 per group (F-H). *p < 0.05, **p < 0.01, and ***p < 0.001; ns, not significant. Unpaired t test (A-B, E-H) was used for statistical analysis.

Combined with our prior findings (DEGs were primarily enriched in mitochondria-related proteins part, which were related to Angiotensin-(1-7)/Mas recptor pathway^21^, ^22^ and synaptic function^23^, ^24^, respectively), we sought to explore the relationship between Angiotensin-(1-7)/MasR pathway and synaptic function. We further experimentally verified the decreased levels of ANG-(1-7) and MasR in the hippocampal region of mice with diabetic cognitive impairment, accompanied by reduced PSD 95 protein levels (**Figure 3A-B**). Immunofluorescence staining demonstrated the strongest expression of Mas receptors in neurons of the hippocampal CA3 subregion (**Figure 3C-D**). We further observed a decreased expression of MasR in the hippocampal CA3 subregion of diabetic mice compared to control mice (**Figure 3E**). Additionally, in primary hippocampal neuron cells from C57BL/6 mice, ELISA and western blot results indicated decreased ANG-(1-7) levels in the cell culture supernatant, along with decreased protein levels of Mas and PSD 95, under high glucose (HG, 75 mM) medium compared to negative control (NC, 25 mM) medium (**Figure 3F-G**). Immunofluorescence staining showed reduced puncta of synapsin I in primary hippocampal neuron cells under high glucose stimulation (**Figure 3H**). These findings suggest that the downregulation of central ANG-(1-7)/MasR may contribute to synaptic damage in diabetic cognitive impairment.

### The Mas agonist AVE 0991 alleviates synaptic damage and improves diabetic cognitive dysfunction

**Figure 4:**
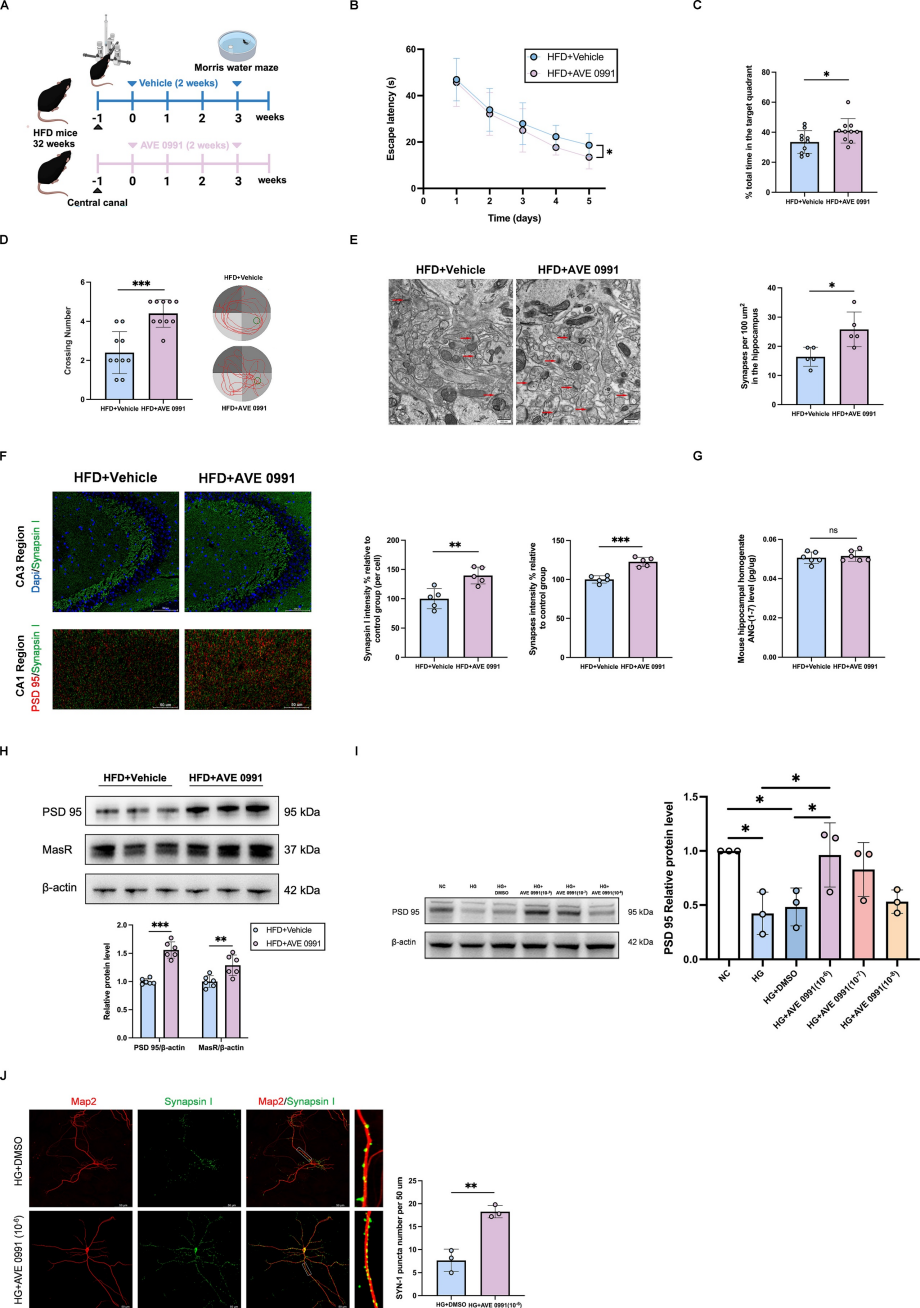
** The Mas agonist AVE 0991 alleviates synaptic damage and improves diabetic cognitive dysfunction.** (A) Experimental scheme. (B-D) Time to find the hidden platform (escape latency), the percentage of time spent in the target quadrant out of the total test time, the numbers of platform crossings numbers, and route map on the last day in the Morris water maze for Vehicle-treated diabetic cognitive impairment mice (HFD+Vehicle) and AVE 0991-treated diabetic cognitive impairment mice (HFD+AVE 0991). (E) Left: Representative electron microscopy of the synaptic structures in mouse hippocampus. Arrows indicate the synapses. Scale bar, 0.5 um. Right: Quantification of synaptic density in mouse hippocampus. (F) Up: representative confocal images of hippocampal immunostaining for Synapsin I (green) in hippocampus CA3 regions. Scale bars, 100 um. Down: representative confocal images depict synaptic staining for pre-synaptic marker Synapsin I (green) and post-synaptic marker PSD95 (red) in hippocampus CA1 regions. Scale bars, 50 um. Right: relative level of synaptic density. (G) Angiotensin-(1-7) levels in mouse hippocampal tissue homogenate between HFD+Vehicle mice and HFD+AVE 0991 mice. (H) MasR and PSD 95 relative protein expression levels in mouse hippocampal brain tissue between HFD+Vehicle group and HFD+AVE 0991 group. (I) PSD 95 relative protein expression levels in primary hippocampal neuron cells treated with NC (25mM), HG (75mM), HG+DMSO, or HG+AVE 0991 (concentrations of 10^-6^, 10^-7^, 10^-8^ M). (J) Left: representative confocal images of MAP2 (red) and Synapsin I (green) in primary hippocampal neuron cells treated with HG+DMSO or HG+AVE 0991 (10^-6^ M). Right: quantification of Synapsin I-positive puncta per 50 um of MAP2-positive neurites. Scale bars, 50 um. Data are presented as the mean ± SD. n = 10 mice/group (B-D), n = 6 per group (G-H), n = 5 per group (E-F), n = 3 per group (I-J). *p < 0.05, **p < 0.01, and ***p < 0.001; ns, not significant. Unpaired t test (C-H, J), one-way ANOVA (I), or two-way ANOVA (B) were used for statistical analysis.

The Mas agonist AVE 0991 (a non-peptide analogue of ANG-(1-7) with a prolonged half-life time and highly specific binding to Mas) 25, was administered to diabetic cognitive impairment mice for two weeks by injecting into the hippocampus using a stereotactic animal brain locator combined with a cannula administration technique (**Figure 4A**). MWM behavioral test results indicated that, compared with vehicle-treated diabetic cognitive impairment mice, AVE 0991-treated diabetic cognitive impairment mice exhibited shorter escape latencies in the hidden platform test, and higher number of platform crossings and longer time in the target quadrant in the probe trial (**Figure 4B-D**). TEM results demonstrated an increased number of synapses in the hippocampal region of AVE 0991-treated diabetic cognitive impairment mice (**Figure 4E**). Additionally, Immunofluorescence staining indicated that AVE 0991-treated diabetic cognitive impairment mice had increased hippocampal synapse numbers compared with Vehicle-treated diabetic cognitive impairment mice (**Figure 4F**). There were no significant differences between AVE 0991-treated diabetic cognitive impairment mice and Vehicle-treated diabetic cognitive impairment mice in terms of hippocampal ANG-(1-7) levels (Figure 4G). Western blot results showed increased expressions of MasR and PSD 95 in the hippocampal region of AVE 0991-treated mice (**Figure 4H**). Furthermore, western blot analysis of primary hippocampal neuron cells revealed an increased PSD 95 protein level under AVE 0991-intervention high glucose stimulation cells, in a concentration-dependent manner, compared to DMSO-intervention high glucose stimulation cells (**Figure 4I**). Immunofluorescence staining displayed increased puncta of synapsin I under AVE 0991-intervention high glucose stimulation cells (**Figure 4J**). In addition, AVE 0991 was administered to Chow-diet mice via injection into the hippocampus for two weeks (**Supplementary Figure 1A**). AVE0991 had no effect on the cognitive performance or hippocampal synapse density in Chow-fed mice (**Supplementary Figure 1B-G**). These findings collectively underscore the efficacy of Mas agonist AVE 0991 in alleviating synaptic damage and improving diabetic cognitive dysfunction.

### FOXO1 is a downstream transcription factor of ANG-(1-7)/MasR that modulates the expression of target gene PACAP and regulates synaptic function

**Figure 5:**
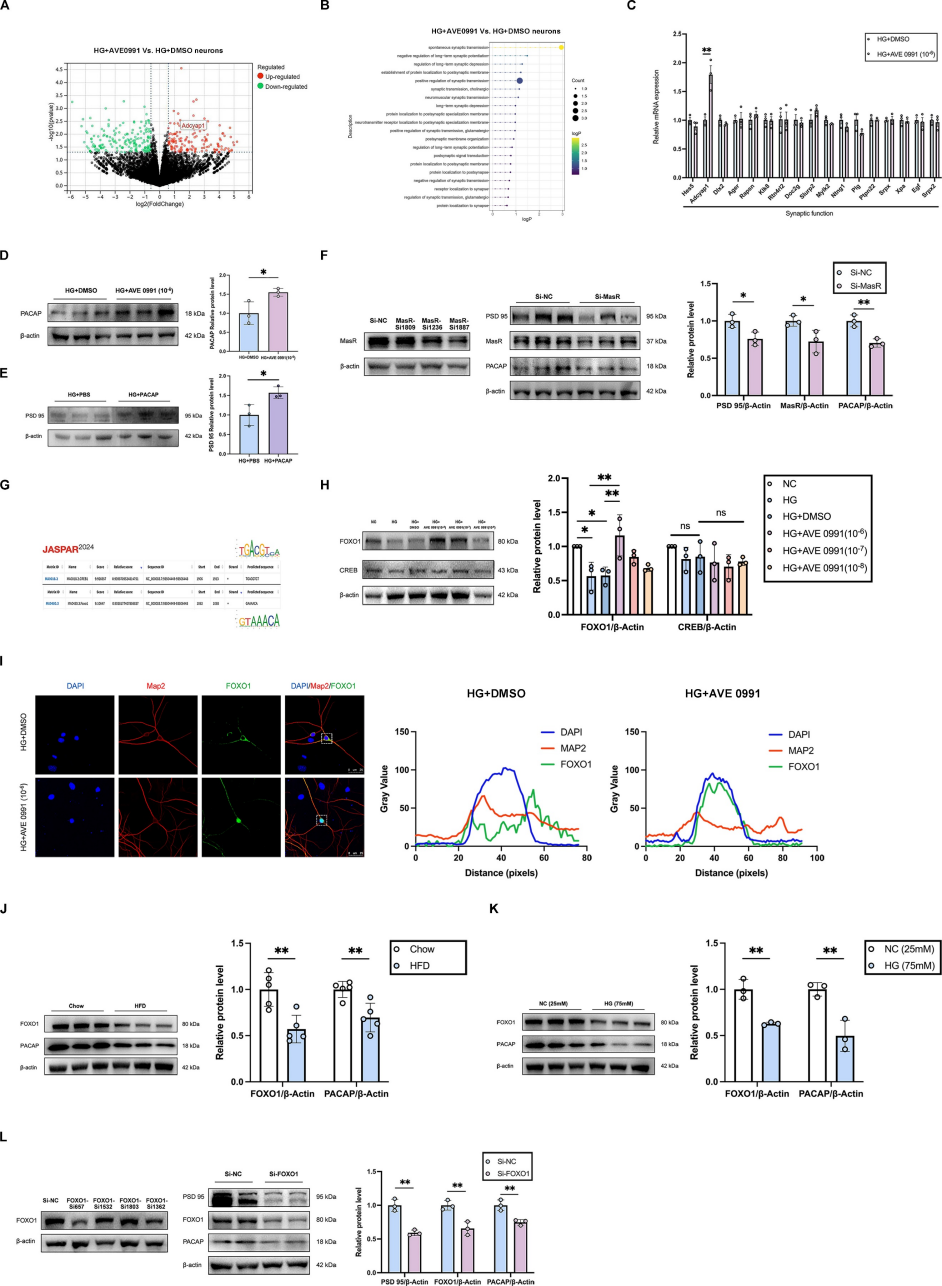
** FOXO1 is a downstream transcription factor of ANG-(1-7)/MasR to modulate the expression of target gene PACAP and regulate synaptic function.** (A) Differential gene volcano map of RNA sequencing in primary hippocampal neuron cells treated with HG+AVE 0991 (10^-6^ M) or HG+DMSO. (B) Lollipop Chart of top 20 enriched synaptic related functions from RNA sequencing data in primary hippocampal neuron cells treated with HG+AVE 0991 (10^-6^ M) or HG+DMSO. (C) Quantitative RT-PCR of genes related to synaptic functions in primary hippocampal neuron cells treated with HG+DMSO or HG+AVE 0991 (10^-6^ M). (D) PACAP relative protein expression levels in primary hippocampal neuron cells treated with HG+DMSO or HG+AVE 0991 (10^-6^ M). (E) PSD 95 relative protein expression in primary hippocampal neuron cells treated with HG+PBS or HG+PACAP. (F) Left: screening for the sequences of different MasR siRNA; Right: PSD 95, MasR and PACAP relative protein expression levels in primary hippocampal neuron cells transfected with Si-MasR or Si-NC. (G) Prediction of downstream transcription factors CREB and FOXO1 of ANG-(1-7)/MasR binding to the PACAP promoter region in the JASPAR database (with score threshold ≥ 90%). (H) CREB and FOXO1 relative protein expression levels in primary hippocampal neuron cells treated with NC (25mM), HG (75mM), HG+DMSO, or HG+AVE 0991 (concentrations of 10^-6^, 10^-7^, 10^-8^ M). (I) Left: Immunostaining of the transcription factors FOXO1 localization in primary hippocampal neuron cells treated with HG+DMSO or HG+AVE 0991 (10^-6^ M). Scale bars, 25 um. Right: The pixel intensity along the distance of the selected area. (J) PACAP and FOXO1 relative protein expression levels in HFD mice and Chow mice. (K) PACAP and FOXO1 relative protein expression levels in primary hippocampal neuron cells cultured with NC (25mM) medium or HG (75mM) medium. (L) Left: screening for the sequences of different FOXO1 siRNA; Right: PSD 95, FOXO1 and PACAP relative protein expression levels in primary hippocampal neuron cells transfected with Si-FOXO1 or Si-NC. Data are presented as the mean ± SD. n = 3 per group (A-F, H-L). *p < 0.05, and **p < 0.01; ns, not significant. Unpaired t test (C-F, J-L), or one-way ANOVA (H) were used for statistical analysis.

RNA sequencing analyses, as well as the validations by qRT-PCR and western blot from the primary hippocampal neuron cells revealed a significant increase in PACAP expression in AVE 0991-intervention high glucose stimulation cells compared with DMSO-intervention high glucose stimulation group (**Figure 5A, C, D**). Additionally, western blot results demonstrated decreased PACAP protein levels in diabetic cognitive impairment mice hippocampus and high glucose-treated primary hippocampal neurons cells (**Figure 5J-K**). Enrichment analysis showed that DEGs were primarily enriched in synaptic function (**Figure 5B**). Western blot results indicated an increased protein level of PSD 95 in PACAP-treated primary hippocampal neuron cells under high glucose stimulation (**Figure 5E**). MasR siRNA inhibited PACAP and PSD 95 protein expression in primary hippocampal neurons (**Figure 5F**). Using the JASPAR database with a score threshold ≥ 90%, we predicted that CREB and FOXO1 may be the downstream transcription factors of Mas, binding to the PACAP promoter region (**Figure 5G**). Further, western blot validation revealed that FOXO1 protein level was reduced in primary hippocampal neurons under high glucose stimulation (75 mM) compared to control medium (25 mM). Conversely, FOXO1 protein level was increased in AVE 0991-intervention high glucose stimulation cells compared to DMSO-intervention high glucose stimulation cells in a concentration-dependent manner (**Figure 5H**). There was no significant difference in CREB protein expression (**Figure 5H**). Immunofluorescence results demonstrated increased FOXO1 nucleus accumulation in primary hippocampal neurons under AVE 0991-intervention high glucose stimulation, indicating enhancement of FOXO1 transcriptional activity (**Figure 5I**). Western blot results indicated decreased FOXO1 protein levels in diabetic cognitive impairment mice hippocampus and high glucose-treated primary hippocampal neurons primary hippocampal neuron cells (**Figure 5J-K**). Furthermore, FOXO1 siRNA inhibited the protein expression of PACAP and PSD 95 in primary hippocampal neurons (**Figure 5I**). These findings suggest that PACAP is a downstream target gene that regulates synaptic function in neuronal cells, and FOXO1 is a downstream transcription factor of ANG-(1-7)/MasR that modulates PACAP expression.

### The AKT signaling pathway acts as a critical upstream regulator of FOXO1 activity in modulating PACAP expression

**Figure 6:**
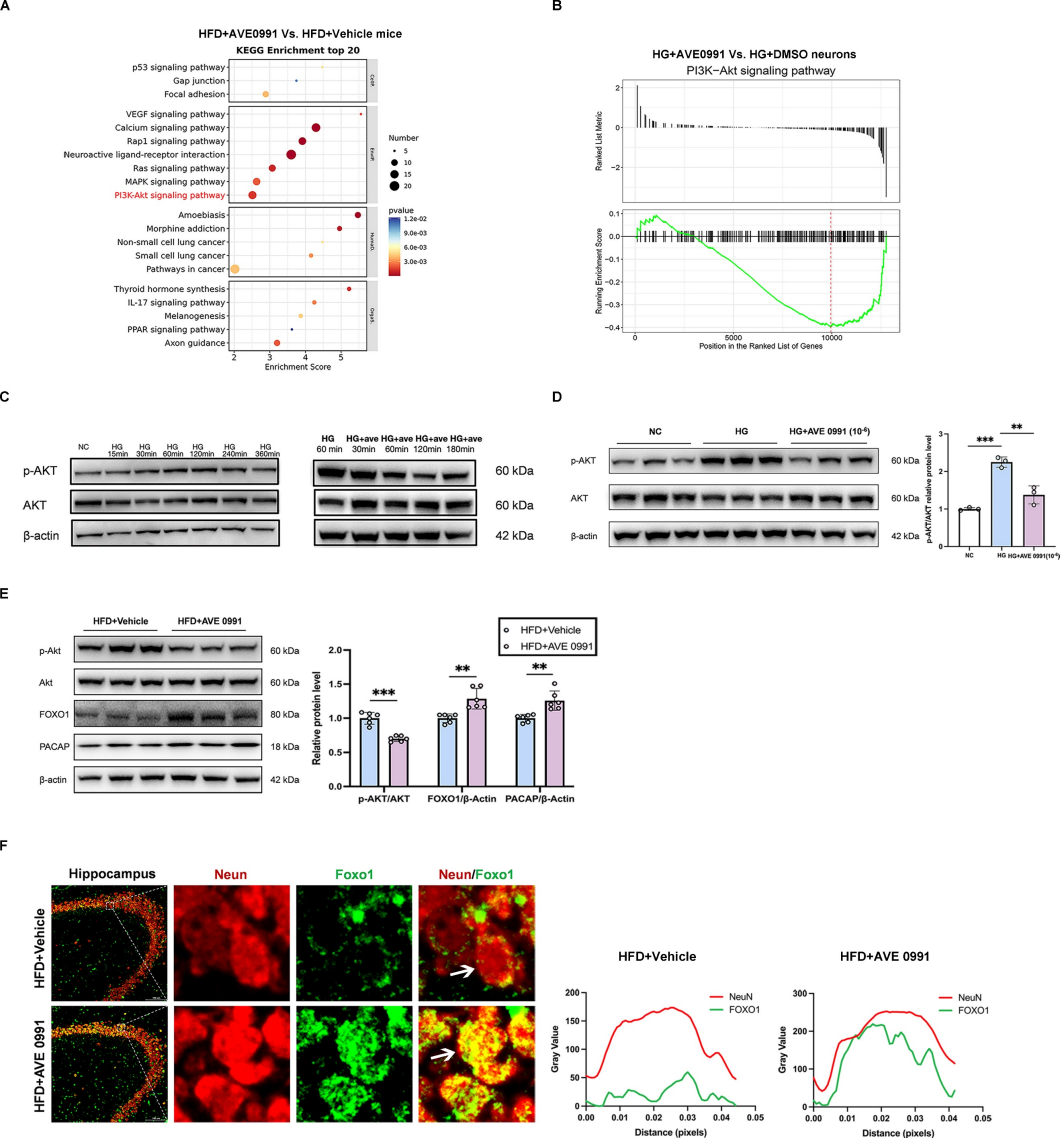
** The AKT signaling pathway acts as a critical upstream regulator of FOXO1 activity in modulating PACAP expression.** (A) KEGG Enrichment analysis of RNA sequencing data from mouse hippocampal tissue between HFD+AVE 0991 group and HFD+Vehicle group. (B) Gene Set Enrichment Analysis (GSEA) of RNA sequencing in primary hippocampal neuron cells treated with HG+AVE 0991 or HG+DMSO. (C) Changes in phosphorylated (p)-Akt and AKT relative protein expression levels with HG or HG+AVE 0991 intervention time. (D) p-Akt and AKT relative protein expression levels in primary neuronal cells treated with NC (25mM), HG (75mM), or HG+AVE 0991 (10^-6^ M). (E) FOXO1, PACAP, p-Akt and AKT relative protein expression levels in mouse hippocampal brain tissue between HFD+Vehicle group and HFD+AVE 0991 group. (F) Left: Immunostaining of the transcription factors FOXO1 localization in the CA3 subregion of the mouse hippocampus between HFD+Vehicle group and HFD+AVE 0991 group. Scale bars, 100 um. Right: The pixel intensity along the distance of the selected area. Data are presented as the mean ± SD. n = 3 per group (A-D), n = 6 mice/group (E), n = 5 per group (F). **p < 0.01, and ***p < 0.001. Unpaired t test (E), or one-way ANOVA (D), were used for statistical analysis.

The KEGG pathway enrichment analysis of our RNA sequencing data revealed a significant downregulation of the PI3K-AKT signaling pathway in AVE 0991-treated diabetic cognitive impairment mice compared with Vehicle-treated diabetic cognitive impairment mice (**Figure 6A**). Furthermore, Gene Set Enrichment Analysis (GSEA) demonstrated a significant downregulation of the PI3K-AKT signaling pathway in primary hippocampal neurons under AVE 0991-intervention high glucose stimulation (**Figure 6B**). Further validation indicated an increased phosphorylated AKT (p-AKT) protein level in primary hippocampal neurons under high glucose (HG, 75 mM) medium compared to negative control (NC, 25 mM) medium, while was reduced in AVE 0991-intervention high glucose stimulation cells compared to DMSO-intervention high glucose stimulation cells (**Figure 6C-D**). Further animal experiments confirmed that compared with Vehicle-treated diabetic cognitive impairment mice, AVE 0991-treated diabetic cognitive impairment mice exhibited reduced p-AKT protein levels, and increased protein levels of FOXO1 and PACAP (**Figure 6E**). Immunofluorescence results indicated an increase of FOXO1 nucleus accumulation in AVE 0991-treated diabetic cognitive impairment mice, suggesting the enhancement of FOXO1 transcriptional activity (**Figure 6F**). These findings suggest that AKT signaling is a critical upstream regulatory pathway for FOXO1 activity in modulating PACAP expression.

### Inhibition of hippocampal neuronal MasR aggravated synaptic damage by regulating the expressions of AKT/FOXO1/PACAP

**Figure 7:**
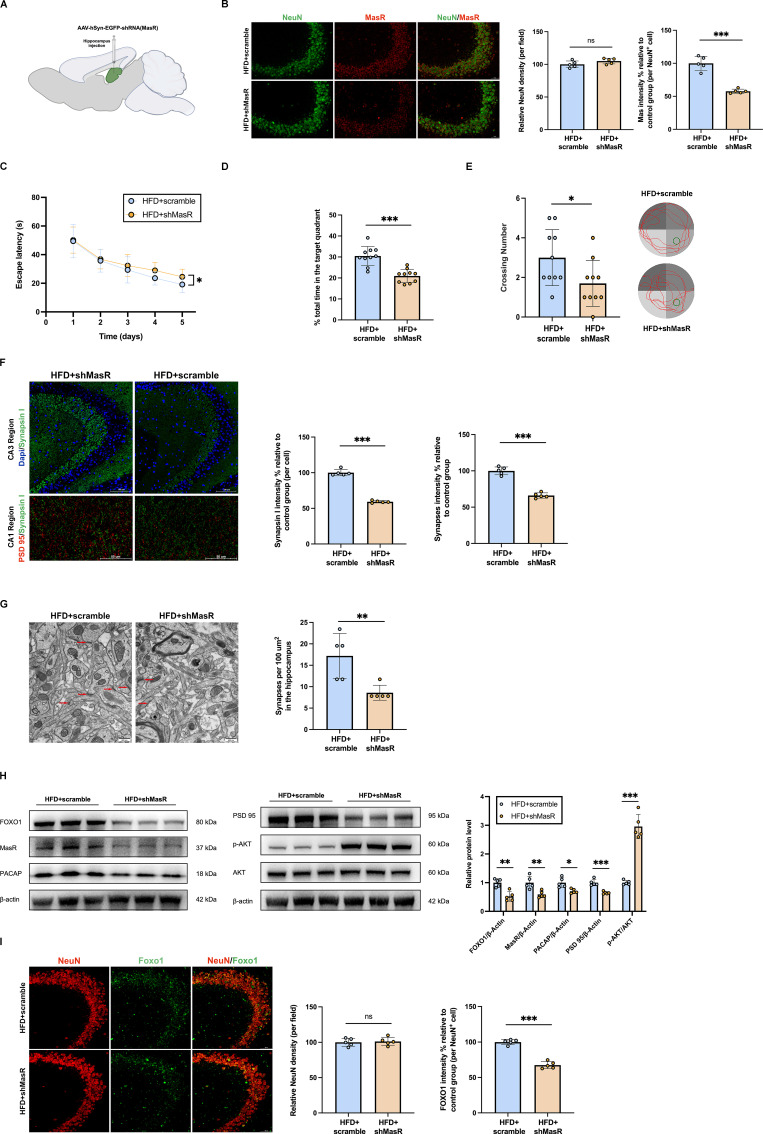
** Inhibition of hippocampal neuronal MasR aggravated synaptic damage by regulating the expressions of AKT/FOXO1/PACAP.** (A) Experimental scheme. (B) Left: representative confocal images of MasR and NeuN in hippocampus 4 weeks after injection. Scale bar, 50 μm. Right: relative level of MasR in hippocampal neurons. (C-E) Time to find the hidden platform (escape latency), the percentage of time spent in the target quadrant out of the total test time, the numbers of platform crossings numbers, and route map on the last day in the Morris water maze for scramble-treated diabetic cognitive impairment mice (HFD+scramble) and AAV-hSyn-EGFP-shRNA (MasR) treated diabetic cognitive impairment mice (HFD+shMasR). (F) Up: representative confocal images of hippocampal immunostaining for pre-synaptic marker Synapsin I (green) in hippocampus CA3 regions. Scale bars, 100 um. Down: representative confocal images depict synaptic staining for pre-synaptic marker Synapsin I (green) and post-synaptic marker PSD 95 (red) in hippocampus CA1 regions. Scale bars, 50 um. Right: relative level of synaptic density. (G) Left: Representative electron microscopy of the synaptic structures in mouse hippocampus. Arrows indicate the synapses. Scale bar, 0.5 um. Right: Quantification of synaptic density in mouse hippocampus. (H) Relative protein expression levels of MasR, p-Akt, AKT, FOXO1, PACAP and PSD 95 in mouse hippocampal brain tissue between HFD+scramble group and HFD+shMasR group. (I) Left: Immunostaining of the transcription factors FOXO1 in the CA3 subregion of the mouse hippocampus between HFD+scramble group and HFD+shMasR group. Scale bars, 50 um. Right: relative level of FOXO1 in hippocampal neurons. Data are presented as the mean ± SD. n = 10 mice/group (B-E), n = 5 per group (F-I). *p < 0.05, **p < 0.01, and ***p < 0.001; ns, not significant. Unpaired t test (B, D-I), or two-way ANOVA (C) were used for statistical analysis.

MasR was knocked down in hippocampal neurons by injecting AAV expressing shRNA against neuronal MasR into the hippocampus of diabetic cognitive impairment mice. MasR protein levels were significantly reduced in the hippocampus of AAV-hSyn-EGFP-shRNA (MasR) treated diabetic cognitive impairment mice (**Figure 7B,H**). The inhibition of hippocampal neuronal MasR aggravated the cognitive dysfunction, and reduced hippocampal synapse density (**Figure 7C-G**). Further experiments confirmed that compared with scramble-treated diabetic cognitive impairment mice, AAV-hSyn-EGFP-shRNA (MasR) treated mice had increased AKT phosphorylation levels, and reduced protein levels of FOXO1, PACAP and PSD 95 (**Figure 7H**). Immunofluorescence results revealed decreased FOXO1 outside the nucleus in AAV-hSyn-EGFP-shRNA (MasR)-treated diabetic cognitive impairment mice, suggesting an additional decrease in FOXO1 transcriptional activity (**Figure 7I**). These results further clarify that AKT/FOXO1/PACAP pathway is the downstream of MasR in regulating synaptic function.

## Discussion

**Figure 8:**
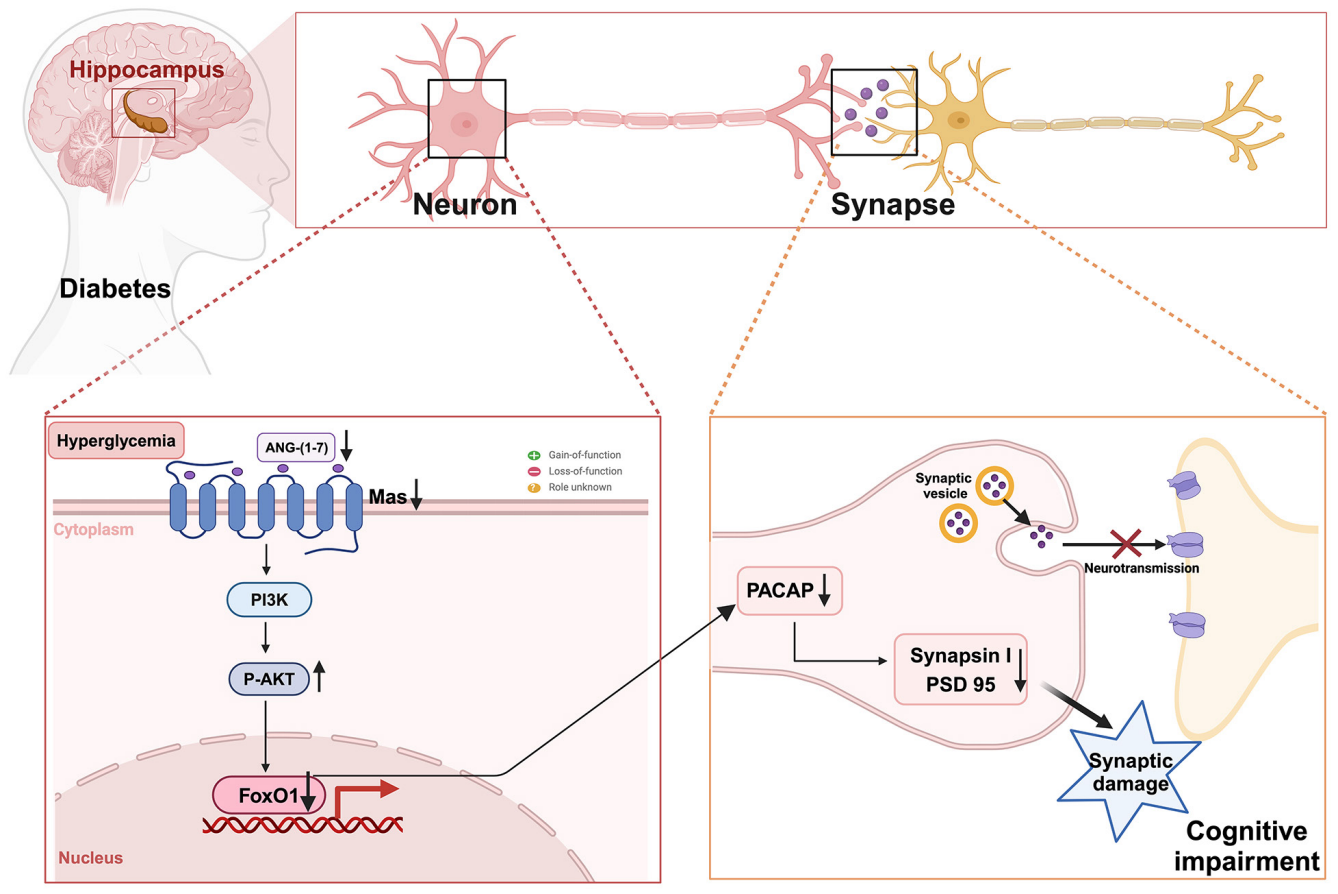
Research hypothesis diagram: The downregulation of ANG-(1-7)/Mas is implicated in synaptic damage, and inhibits synaptic function-related target gene PACAP expression through the AKT/FOXO1 signaling pathway in diabetes-associated cognitive impairment.

In this study, we demonstrated for the first time that the downregulation of ANG-(1-7)/MasR plays a pivotal role in synaptic and memory dysfunction in DACI. We observed a significant reduction in ANG-(1-7) and MasR levels in the hippocampal region of HFD-induced diabetic mice. Notably, serum ANG-(1-7) levels were also decreased in the T2DM-MCI patients. Administration of the Mas agonist AVE 0991 into the hippocampus ameliorated synaptic and memory dysfunctions in diabetic cognitive impairment mice. Mechanistically, we first elucidated that PACAP is a downstream target gene of ANG-(1-7)/MasR that regulates synaptic function. Furthermore, we identified FOXO1 as an important downstream transcription factor of ANG-(1-7)/MasR in modulating the expression of target gene PACAP, by directly binding to the PACAP promoter region. We also observed decreased levels of phospho-Akt, a negative regulator of FOXO1 activity, after AVE 0991 treatment both in vitro and in vivo experiments. Therefore, our data suggest that MasR/AKT/FOXO1/PACAP may be potential intervention targets for DACI. A schematic diagram of our research hypothesis is depicted in **Figure 8**.

Recent studies suggest that hippocampal synaptic plasticity forms the foundation of learning and memory 3-5. Synaptic proteins, including synapsin I, PSD 95, and Homer-1, contribute to synaptic transmission 26-28. Previous investigations have demonstrated a proportional relationship between reduced synaptic proteins and synaptic damage, which results in impaired cognition and information transmission and cognitive impairment 5, 29. Our RNA-seq data revealed that DEGs were mainly enriched in axon part, postsynaptic density, and neuron to neuron synapse. We also observed synaptic damage and memory impairment in diabetic cognitive impairment mice. Furthermore, in vitro experiments demonstrated decreased synaptic protein expression in primary hippocampal neuron cells under high glucose stimulation. All these results indicate that high glucose can induce synaptic damage and memory impairment.

The recptor Mas, a G protein-coupled receptor encoded by the oncogene Mas1, is highly expressed in the brain and selectively binds to ANG-(1-7) 14, 15. MasR has been reported to be primarily distributed in neurons, astrocytes, and microglia of the hippocampus, cortex, and basal ganglia regions 15, 30. Given the widespread distribution of MasR, it is crucial to investigate Mas signaling pathways in a cell-type-specific manner. Consistent with previous studies mentioned above, our data show that Mas receptors are most strongly expressed in neurons in the CA3 region of the mouse hippocampus. Furthermore, we confirmed that ANG-(1-7)/MasR levels were reduced in T2DM-MCI patients, diabetic cognitive impairment mice, and primary hippocampal neuron cells under high glucose stimulation. AVE 0991, a non-peptide analogue of ANG-(1-7), has a prolonged half-life time and is highly specific binding to Mas receptor 25, 31. In AVE 0991-treated diabetic cognitive impairment mice, we found that hippocampal ANG-(1-7) levels were unchanged, but hippocampal MasR protein levels were increased. We speculate that the restoration of hippocampal MasR level is an adaptive change caused by the exogenous administration of AVE 0991 to activate the Mas receptor. Synaptic protein levels and synaptic numbers were increased following the treatment with AVE 0991 both in vivo and in vitro, while synaptic protein levels were decreased after MasR siRNA transfection in primary hippocampal neurons. The knock down of hippocampal neuronal MasR in diabetic cognitive impairment mice aggravated cognitive dysfunction, and reduced hippocampal synapse density and PSD 95 protein levels. These results confirm the important role of MasR in modulating synaptic function in neurons.

We further investigated the mechanisms and target molecules involved in the MasR-mediated regulation of neuronal synaptic function. Our RNA-seq analyses revealed an upregulation of pituitary adenylate cyclase-activating polypeptide (PACAP) among DEGs associated with synaptic function regulation. Additionally, enrichment analysis indicated a positive regulatory effect on synaptic function. PACAP, encoded by the ADCYAP1 gene, is a neuropeptide with diverse biological functions, including neurotrophic and neuromodulatory effects 32-38. Furthermore, evidence from previous studies suggests that PACAP can enhance cognitive abilities by inhibiting the deposition of Aβ and Tau, and promoting the clearance of Tau and Aβ aggregates in Alzheimer's disease mice 39, 40. PACAP also exhibits anti-apoptotic effects in a hypoperfusion model and modulates synaptic plasticity through Sirt3 41. Research has shown that synaptic proteins such as PSD 95 can reflect synaptic function, synaptic plasticity, and cognitive level 42. Consistent with a previous study 41, we found that PACAP activation can increase the level of PSD 95 in vitro. In this study, we demonstrated increased PACAP levels following the treatment with AVE 0991 both in vivo and in vitro. However, PACAP levels were decreased under high glucose stimulation both in vivo and in vitro, as well as decreased after MasR siRNA transfection in vitro and shMasR inhibition in vivo. These findings indicate that PACAP serves as a downstream target gene of MasR in regulating synaptic function.

FOXO1 is a transcription factor that plays a crucial role in cellular metabolism, development, aging, and longevity 43-45. Previous studies have found that ANG-(1-7) directly activates FOXO1 signaling through Mas, regulating FOXO1 translocation and downstream gene transcription 10, 46, 47. Consistent with these findings, we observed an increase in FOXO1 levels and nucleus accumulation under AVE 0991 intervention in vivo and in vitro, indicating enhancement in FOXO1 transcriptional activity. However, FOXO1 levels were decreased under high glucose stimulation both in vivo and in vitro, and FOXO1 outside the nucleus were decreased after shMasR inhibition in vivo, suggesting a further decrease in FOXO1 transcriptional activity. Previous evidence has shown that FOXO transactivates genes involve in neurogenesis, inflammation, oxidative stress, and autophagy, which are crucial for maintaining brain homeostasis 10, 48, 49. Notably, recent studies have also found that FOXO deficiency results in abnormal dendritic morphology 50. Using the JASPAR database, we predicted that FOXO1, as a downstream transcription factors of MasR, would bind to the PACAP promoter region. Furthermore, we confirmed that PACAP and PSD 95 levels were decreased after FOXO1 siRNA transfection in primary hippocampal neurons. Together, these results demonstrate that FOXO1 is an important downstream transcription factor of ANG-(1-7)/MasR in modulating the expression of target gene PACAP, and regulating synaptic function.

It is well known that AKT negatively regulates FOXO1 through phosphorylation, thereby preventing FOXO1 nuclear accumulation and impairing target gene regulation 51-53. Our RNA-seq KEGG pathway enrichment analysis and GSEA analysis, both revealed a downregulation of the PI3K-AKT signaling pathway. Further validation in vivo and in vitro demonstrated that MasR activation in neurons is accompanied by reduced AKT phosphorylation levels, increased FOXO1 levels, and enhanced FOXO1 nuclear accumulation. In addition, AKT phosphorylation levels were increased and FOXO1 levels were reduced in knock down of hippocampal neuronal MasR in diabetic cognitive impairment mice. It can be seen that, the negative regulation relationships between AKT and FOXO1, downstream effectors of ANG-(1-7)/MasR signaling, still exist in neurons. These findings greatly enhance our understanding of the molecular mechanisms by which MasR regulates synaptic function-related target gene PACAP.

In conclusion, our study elucidated that the downregulation of ANG-(1-7)/MasR is implicated in synaptic damage in DACI. MasR regulates synaptic function-related target gene PACAP expression through the AKT/FOXO1 signaling pathway, thereby providing a deeper theoretical basis and molecular target for the future treatments of DACI.

## Supplementary Material

Supplementary figure and table.

## Figures and Tables

**Table 1:** Demographic and clinical data of the study individuals.

Characteristics	T2DM-non MCI (n = 69)	T2DM-MCI (n = 51)	P value
Demographic factors			
Age (years)	59.64 ± 0.94	61.16 ± 0.94	0.267^a^
Female sex (n, %)	25 (36.23%)	20 (39.22%)	0.739^c^
BMI (kg/m2)	24.78 ± 0.40	24.30 ± 0.40	0.414^a^
WHR	0.91 ± 0.01	0.91 ± 0.01	0.425^b^
Education (years)	13.04 ± 0.38	12.31 ± 0.42	0.169^b^
Diabetes duration(years)	12.45 ± 0.75	14.92 ± 1.10	0.125^b^
SBP (mmHg)	136.06 ± 2.23	134.35 ± 2.83	0.632^a^
DBP (mmHg)	84.87 ± 1.35	82.10 ± 1.61	0.187^a^
Metabolic factors			
FBG (mmol/L)	7.78 ± 0.29	8.33 ± 0.31	0.044^b,*^
FINS (μIU/mL)	7.50 ± 0.88	10.78 ± 1.70	0.259^b^
FCP (pmol/L)	614.35 ± 42.01	586.86 ± 47.01	0.669^b^
HbA1c (%)	8.17 ± 0.23	8.19 ± 0.24	0.625^b^
HOMA-IR	2.75 ± 0.38	7.80 ± 3.28	0.107^b^
Serum creatinine (umol/L)	60.58 ± 1.83	63.65 ± 2.56	0.451^b^
Serum urea (mmol/L)	5.80 ± 0.17	5.78 ± 0.20	0.899^b^
Serum uric acid (umol/L)	321.26 ± 9.17	340.86 ± 10.85	0.175^b^
TG (mmol/L)	1.64 ± 0.15	1.63 ± 0.13	0.780^b^
TC (mmol/L)	4.85 ± 0.19	4.66 ± 0.16	0.774^b^
HDL-c (mmol/L)	1.26 ± 0.04	1.27 ± 0.05	0.880^b^
LDL-c (mmol/L)	2.91 ± 0.15	2.77 ± 0.13	0.712^b^
ApoA1(g/L)	1.20 ± 0.03	1.22 ± 0.03	0.639^a^
ApoB(g/L)	0.91 ± 0.04	0.88 ± 0.04	0.627^a^
C-reactive protein	4.17 ± 1.12	5.26 ± 1.25	0.460^b^
ANG-(1-7) (ng/mL)	2.48 ± 0.07	2.19 ± 0.08	0.009^a,**^
Cognition test levels			
MoCA	27.46 ± 0.15	23.14 ± 0.25	< 0.001^b,***^

Abbreviations: MCI, mild cognitive impairment; BMI, body mass index; WHR, Waist-to-Hip Ratio; SBP, systolic blood pressure; DBP, diastolic blood pressure; FBG, fasting blood glucose; FINS, fasting insulin; FCP, fasting c-peptide; HbAlc, glycosylated hemoglobin; HOMA-IR, the homeostasis model assessment of insulin resistance; TG, triglyceride; TC, total cholesterol; HDL-c, high-density lipoprotein cholesterol; LDL-c, low-density lipoprotein cholesterol; ANG-(1-7), Angiotensin-(1-7); Data were expressed as Data are presented as n (%), mean ± SEM, or median (interquartile range) as appropriate. *p < 0.05, **p < 0.01, and ***p < 0.001. Data are presented as n (%), mean ± SEM, or median (interquartile range) as appropriate. a Student's t-test for comparison of normally distributed quantitative variables between T2DM-MCI group and T2DM-non MCI group. b Mann-Whitney U test for comparison of asymmetrically distributed quantitative variables between T2DM-MCI group and T2DM-non MCI group. c χ^2^ test for comparison of qualitative variables between T2DM-MCI group and T2DM-non MCI group.

**Table 2:** Hippocampal subfield volumes (mm^3^) of the study individuals.

	T2DM-non MCI (n = 22)	T2DM-MCI (n = 16)	P value
L-Whole hippocampal	3549.84 ± 66.09	3407.73 ± 79.83	0.529
L-Subiculum	460.11 ± 9.79	437.14 ± 14.63	0.877
L-Presubiculum	321.39 ± 7.74	294.58 ± 8.51	0.326
L-Parasubiculum	65.72 ± 2.72	66.94 ± 3.47	0.492
L-CA1	651.06 ± 12.58	620.26 ± 20.95	0.617
L-CA3	210.07 ± 5.64	208.97 ± 7.65	0.558
L-CA4	246.48 ± 4.77	239.19 ± 5.68	0.767
L-GC-ML-DG	286.98 ± 5.49	275.48 ± 7.20	0.965
L-Fissure	158.14 ± 5.17	167.44 ± 8.68	0.108
L-Molecular layer	570.11 ± 10.65	538.81 ± 14.39	0.961
L-Fimbria	80.51 ± 3.51	83.63 ± 5.24	0.275
L-HATA	57.15 ± 2.12	53.44 ± 2.20	0.416
R-Whole hippocampal	3678.56 ± 78.07	3505.61 ± 105.42	0.482
R-Subiculum	461.01 ± 10.22	435.91 ± 14.46	0.941
R-Presubiculum	312.45 ± 7.78	275.27 ± 6.73	0.036^*^
R-Parasubiculum	58.71 ± 2.42	63.22 ± 4.48	0.202
R-CA1	698.10 ± 16.53	669.83 ± 27.22	0.457
R-CA3	225.53 ± 6.20	225.33 ± 8.77	0.260
R-CA4	256.28 ± 5.77	249.13 ± 7.19	0.226
R-GC-ML-DG	299.88 ± 6.77	288.42 ± 8.46	0.300
R-Fissure	170.44 ± 5.78	169.87 ± 6.67	0.646
R-Molecular layer	597.15 ± 12.83	566.52 ± 18.93	0.672
R-Fimbria	70.66 ± 3.49	70.62 ± 5.27	0.392
R-HATA	57.40 ± 2.13	53.46 ± 2.47	0.777

Abbreviations: L Left, R Right *p < 0.05, differences in brain structure between T2DM-MCI group and T2DM-non MCI group were compared using analyses of covariance controlling for sex, age, years of education, and total intracranial volume. Data are presented as mean ± SEM, or median (interquartile range) as appropriate.

## Abbreviations

ANG-(1-7): Angiotensin-(1-7)
BMI: body mass index
BSA: bovine serum albumin
CI: confidence intervals
DACI: diabetes-associated cognitive impairment
DBP: diastolic blood pressure
DEG: differentially expressed gene
DTI: diffusion tensor imaging
ELISA: enzyme-linked immunosorbent assay
FA: fractional anisotropy
FBG: fasting blood-glucose
FCP: fasting C peptide
GSEA: Gene Set Enrichment Analysis
HbA1c: glycosylated hemoglobin
HDL-c: high-density lipoprotein cholesterol
HFD: high-fat diet
LDL-c: low-density lipoprotein cholesterol
MAP2: microtubule-associated protein 2
MCI: mild cognitive impairment
MoCA: Montreal Cognitive Assessment
MRI: Magnetic resonance imaging
MWM: Morris water maze
OR: odds ratios
PACAP: pituitary adenylate cyclase-activating polypeptide
PBS: phosphate-buffered saline
PSD 95: postsynaptic density protein 95
SBP: systolic blood pressure
SD: standard deviation
SEM: standard error of mean
T2DM: type 2 diabetes mellitus
TC: total cholesterol
TEM: Transmission electron microscope
TG: triglycerides
TSA: Tyramide Signal Amplification
WHR: waist-to-hip ratio
