# Supplementary material for: Activation of central Angiotensin-(1-7)/Mas receptor alleviates synaptic damage in diabetes-associated cognitive impairment via modulating AKT/FOXO1/PACAP axis
Source: Int J Biol Sci. 2025 Apr 9;21(6):2824–42. doi: 10.7150/ijbs.99617 (PMC12035909; doi:10.7150/ijbs.99617)
Supplement: Supplementary file 1 — Supplementary figure and table. [file ijbsv21p2824s1.pdf]

Supplementary Table 1. Key resources table.

REAGENT or RESOURCE	SOURCE	IDENTIFIER
**Antibodies**		
Neun antibody	Abcam	Cat# ab177487
GFAP antibody	Abcam	Cat# ab68428
Iba-1 antibody	Wako	Cat# 019-19741
MAP2 antibody	Abcam	Cat# ab5392
PSD 95 antibody	Abcam	Cat# ab18258
PSD 95 antibody	Cell Signaling Technology	Cat# 3450
Synapsin I antibody	Millipore	Cat# AB1543
Mas antibody	Santa Cruz	Cat# sc-390453
Mas antibody	Bioworld	Cat# MB11189
FOXO1 antibody	Cell Signaling Technology	Cat# 2880
FOXO1 antibody	Proteintech	Cat# 18592-1-AP
AKT antibody	Cell Signaling Technology	Cat# 4691
phospho-AKT antibody	Cell Signaling Technology	Cat# 4060
β-Actin	Bioworld	Cat# AP0060

**Chemicals**		
AVE 0991	MedChemExpress	Cat# HY-15778
PACAP 1-38	Selleck Chemicals	Cat# S8415
B27 supplement	ThermoFisher Scientific	Cat# 17504-044
Neurobasal medium	Gibco	Cat# 21103-049
L-Glutamine, 200 mM Solution	ThermoFisher Scientific	Cat# 25030081
DMEM/F-12	Gibco	Cat# 11320082
Poly-D-Lysine	Sigma	Cat# P6407
Triton X-100	Roche	Cat# 11332481001
DAPI	Bioworld	Cat# BD 5010
TRIzol reagent	ThermoFisher Scientific	Cat# 15596018
60% high fat diet	Research Diets	Cat# D12492
RIPA buffer	ThermoFisher Scientific	Cat# 89900
**Critical commercial assays**		
PrimeScript™ RT Master Mix	TAKARA	Cat# RR036A
ECL Western Blotting Substrate	Tanon Technologies	Cat# 180-5001
Mouse Angiotensin 1-7(Ang1-7)ELISA	Cusabio Biotech	Cat# CSB-E13763m

Kit		
Human Angiotensin 1-7(Ang1-7)ELISA Kit	Cusabio Biotech	Cat# CSB-E14242h
**Oligonucleotides**		
β-actin forward	GENEray	GTGACGTTGACATCCGTAAAGA
β-actin reverse	GENEray	GCCGGACTCATCGTACTCC
Hes5 forward	GENEray	CCGTCAGCTACCTGAAACACAG
Hes5 reverse	GENEray	GGTCAGGAACTGTACCGCCTC
Adcyap1 forward	GENEray	AGTGTCTCCTGTTCACCTGCCG
Adcyap1 reverse	GENEray	AGTAAAGGGCGTAAGCGTCACG
Dlx2 forward	GENEray	GTCTCCTACTCCGCCAAAAGCA
Dlx2 reverse	GENEray	GGATTTCAGGCTCAAGGTCTTCC
Ager forward	GENEray	GCCACTGGAATTGTCGATGA

		GG
Ager reverse	GENEray	GCTGTGAGTTCAGAGGCAG GAT
Rapsn forward	GENEray	GTGGATGAAGGTGCTGGAG AAG
Rapsn reverse	GENEray	CCGAGCAGTATCAATCTGGA CC
Klk8 forward	GENEray	TCCTGGTTGGAGACAGATG GGT
Klk8 reverse	GENEray	AGGATGCTGGATAGACTGA GCC
Rtn4rl2 forward	GENEray	CCTGGCAACATTTTCCGAGG CT
Rtn4rl2 reverse	GENEray	AGGAAGAGGTGGCTCAGGT TGG
doc2g forward	GENEray	ACACAGCTCGTGGCATGTCT CT
Doc2g reverse	GENEray	GAGCAAGGTGAACACAGCG TAG
Slurp2 forward	GENEray	CAATGCCACCTGTGCAAGG GAT
Slurp2 reverse	GENEray	CAGCCACTGTAGCACATCTT

		CG
Mylk2 forward	GENEray	TACGCAGCCATTGAGACCTC TC
Mylk2 reverse	GENEray	ATGGTGTCCACCTCCGTCAG AT
Ntng1 forward	GENEray	GACCTGAGGATCAGGCTGTT GA
Ntng1 reverse	GENEray	ACACGAAGTGGCATGCAGG TTG
Plg forward	GENEray	CCTCATAGGCACAACAGGA CAC
Plg reverse	GENEray	TGGCTGTCAGTGGTATAGCA CC
Ptpn22 forward	GENEray	CGGTAGAAGCTGACTCTTGT CC
Ptpn22 reverse	GENEray	CCAACTCTTCCTCGGCATTC ATC
Srpx forward	GENEray	CATCTGCCAGTCAAACAAG CGC
Srpx reverse	GENEray	CACCGAGAGTTAAAGTAGG CACC
Xpa forward	GENEray	GAAGAACCCACGCCATTCA

		CAG
Xpa reverse	GENEray	CTCGGTTTTCCTGCCTCACTTC
Egf forward	GENEray	ACTGGTGTGACACCAAGAGGTC
Egf reverse	GENEray	CCACAGGTGATCCTCAAACACG
Srpx2 forward	GENEray	CGTTATACTGCCTATGACCGAGC
Srpx2 reverse	GENEray	CACAGATGGCACCATAGTTGTCC
Si-MasR-1809 sense	GenePharma	GCUUCAGGGAGUCCUUAAATT
Si-MasR-1809 antisense	GenePharma	UUUAAGGACUCCCUGAAGCTT
Si-MasR-1236 sense	GenePharma	CUGGCCAUCACUACACAAUTT
Si-MasR-1236 antisense	GenePharma	AUUGUGUAGUGAUGGCCAGTT
Si-MasR-1887 sense	GenePharma	GCAACACUGUAUCCAUUGATT
Si-MasR-1887	GenePharma	UCAAUGGAUACAGUGUUGC

antisense		TT
Si-FOXO1-1803 sense	GenePharma	GAGGAUUGAACCAGUAUA ATT
Si-FOXO1-1803 antisense	GenePharma	UUAUACUGGUUCAAUCCUC TT
Si-FOXO1-1532 sense	GenePharma	CCCAGUCUGUCUGAAAUCA TT
Si-FOXO1-1532 antisense	GenePharma	UGAUUUCAGACAGACUGGG TT
Si-FOXO1-1362 sense	GenePharma	GCAACGAUGACUUUGAUAA TT
Si-FOXO1-1362 antisense	GenePharma	UUAUCAAAGUCAUCGUUGC TT
Si-FOXO1-657 sense	GenePharma	GCACCGACUUUAUGAGCAA TT
Si-FOXO1-657 antisense	GenePharma	UUGCUCAUAAAGUCGGUGC TT
**Experimental models**		
Mouse: C57BL/6J	NBRI	Cat# N000013
**Deposited data**		
RNA sequence raw data	Novogene	X101SC20011047-Z01

RNA sequence raw data	Oebiotech	ZOE2023010959; DZOE20230777568
**Software and algorithms**		
GraphPad Prism 9.0	Graphpad	RRID: SCR_002798
Fiji-ImageJ	National Inst. Of Health	RRID: SCR_003070
SPSS software (version 26.0)	IBM	RRID: SCR_019096

Supplementary Figure 1. The Mas agonist AVE 0991 in Chow-fed mice had no effect on cognitive performance and hippocampal synaptic density. (related to Figure 4)

(A) Experimental scheme.

(B-D) Time to find the hidden platform (escape latency), the percentage of time spent in the target quadrant out of the total test time, the numbers of platform crossings numbers, and route map on the last day in the Morris water maze for Vehicle-treated Chow-fed mice (Chow+Vehicle) and AVE 0991-treated Chow-fed mice (Chow+AVE 0991).

(E) Left: Representative electron microscopy of the synaptic structures in mouse hippocampus. Arrows indicate the synapses. Scale bar, 0.5 um. Right: Quantification of synaptic density in mouse hippocampus.

(F) Up: representative confocal images of hippocampal immunostaining for pre-synaptic marker Synapsin I (green) in hippocampus CA3 regions. Scale bars, 100 um. Down: representative confocal images depict synaptic staining for pre-synaptic marker Synapsin I (green) and post-synaptic marker PSD 95 (red) in hippocampus CA1 regions. Scale bars, 50 um. Right: relative level of synaptic density.

(G) PSD 95 relative protein expression levels in mouse hippocampal brain tissue between Chow+Vehicle group and Chow+AVE 0991 group.

Data are presented as the mean ± SD. n = 10 mice/group (B-D), n = 5 per group (E-G). *ns, not significant. Unpaired t test (C-G), or two-way ANOVA (B) were used for statistical analysis.

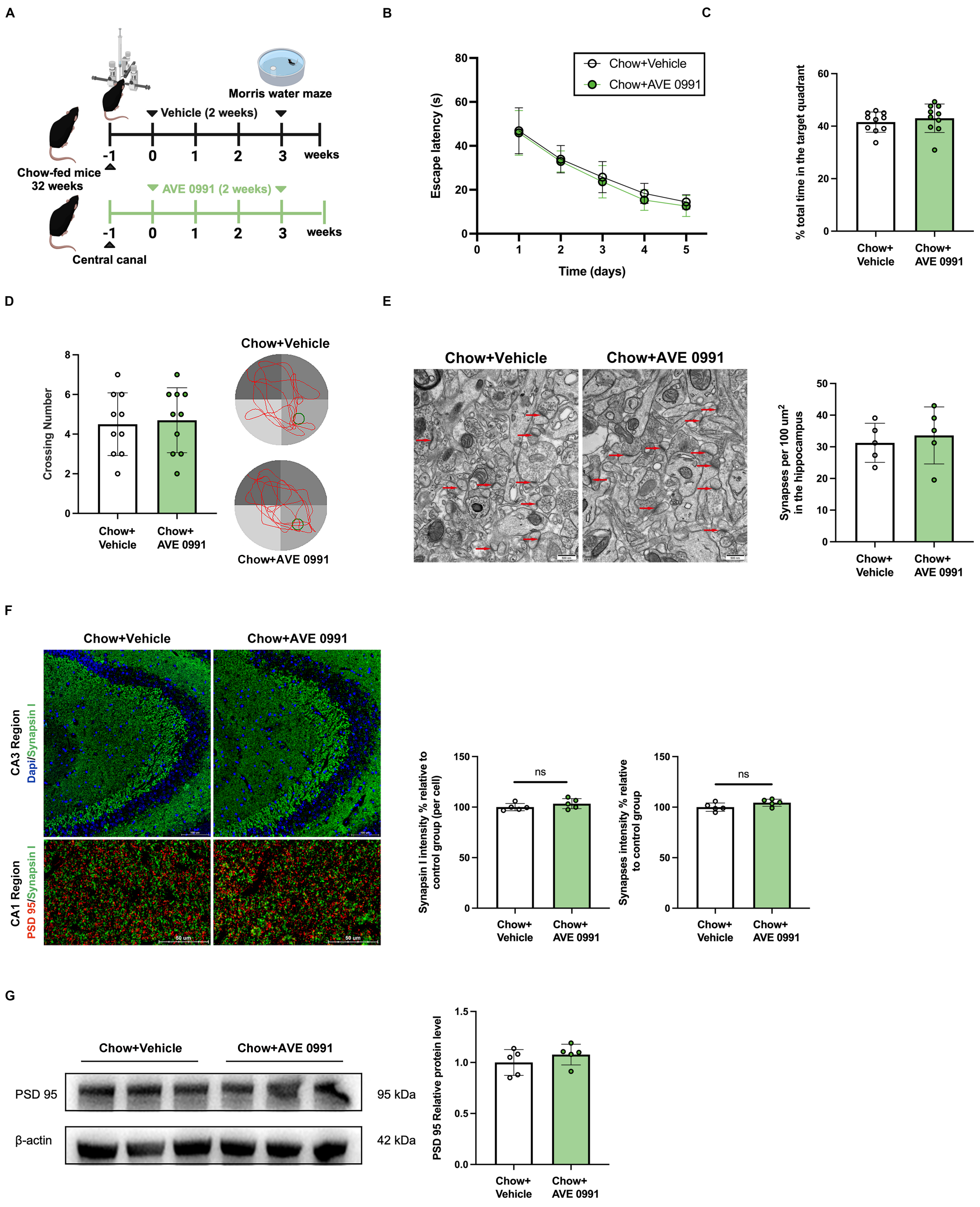

A
Morris water maze
Vehicle (2 weeks)
-1
0
1
2
3
weeks
Chow-fed mice
32 weeks
AVE 0991 (2 weeks)
Central canal
B
Chow+Vehicle
Chow+AVE 0991
Escape latency (s)
Time (days)
C
% total time in the target quadrant
Chow+ Vehicle
Chow+ AVE 0991
D
Crossing Number
Chow+Vehicle
Chow+AVE 0991
E
Chow+Vehicle
Chow+AVE 0991
Synapses per 100 um2 in the hippocampus
F
Chow+Vehicle
Chow+AVE 0991
CA3 Region
Dapi/Synapsin I
CA1 Region
PSD 95/Synapsin I
Synapsin I intensity % relative to control group (per cell)
Synapses intensity % relative to control group
ns
G
Chow+Vehicle
Chow+AVE 0991
PSD 95
β-actin
95 kDa
42 kDa
PSD 95 Relative protein level
